# The Management of Neonates ≥34 Weeks’ Gestation at Risk of Early Onset Sepsis: A Pilot Study

**DOI:** 10.3390/antibiotics12081306

**Published:** 2023-08-10

**Authors:** Maria Cristina Barbini, Simona Perniciaro, Ilia Bresesti, Laura Folgori, Lucia Barcellini, Angela Bossi, Massimo Agosti

**Affiliations:** 1 Neonatal Intensive Care Unit, “Filippo del Ponte” Hospital, ASST Settelaghi, 21100 Varese, Italy; mariacristina.barbini@asst-settelaghi.it (M.C.B.); simona.perniciaro@asst-settelaghi.it (S.P.); angela.bossi@asst-settelaghi.it (A.B.); massimo.agosti@asst-settelaghi.it (M.A.); 2 Department of Medicine and Surgery, University of Insubria, 21100 Varese, Italy; 3 Department of Pediatrics, “V. Buzzi” Children’s Hospital, ASST FBF Sacco, 20154 Milan, Italy; folgori.laura@asst-fbf-sacco.it (L.F.); lucia.barcellini@unimi.it (L.B.)

**Keywords:** early onset sepsis, newborns, antibiotics

## Abstract

Early onset sepsis (EOS) is a potentially fatal condition in neonates, and its correct management is still challenging for neonatologists. Early antibiotic administration in the neonatal period may carry short- and long-term risks. Neonatal EOS calculator has been recently introduced as a new strategy to manage infants at risk of sepsis, and has shown promising results. Methods: In this single-center observational retrospective study, 1000 neonates ≥ 34 weeks’ gestation were enrolled with the aim to evaluate our standard protocol for the management of suspected EOS compared to the EOS calculator. Outcome measures included the following: (1) incidence of EOS and (2) proportion of infants in need of sepsis evaluations and antibiotics using our standard protocol versus theoretical application of EOS calculator. Results: A total of 223/1000 infants underwent blood investigations versus 35/1000 (3.5%) if EOS calculator had been applied (*p* < 0.0001; k = 0.18). Furthermore, 48/1000 infants received antibiotics with our protocol versus 35/1000 with EOS calculator (*p* = 0.12; k = 0.58). Three infants had a positive blood culture that EOS calculator would have missed. Conclusions: In our study, EOS calculator could have reduced investigations but not antibiotic therapy. EOS calculator is an effective and promising tool, but further studies are required to improve it.

## 1. Introduction

Neonatal sepsis is a systemic infection occurring in the first 28 days of life. Although the latest definition refers to early onset sepsis (EOS) as culture-proven sepsis within the first 72 h after birth [1], in the neonatal population a relevant proportion of sepsis is diagnosed based on clinical symptoms [2]. The incidence in high-income countries has significantly decreased over the past two decades, especially with the implementation of evidence-based intrapartum antimicrobial prophylaxis [1]. Currently, with the implementation of universal maternal antenatal screening and intrapartum antibiotic prophylaxis (IAP), the national incidence of group B streptococcus (GBS) EOS has declined from 1.8 cases per 1000 live births in 1990 to 0.23 cases per 1000 live births in 2015 [3].

Approximately 60% of term infants with EOS require intensive medical care, and mortality is around 2% to 3% [4,5,6].

Specific clinical risk factors have been described [7,8]. Moreover, the infant’s clinical condition at birth and the subsequent clinical course over the first 12 to 24 h after delivery are the strongest predictors of early-onset infections [7,9].

**Figure 1:**
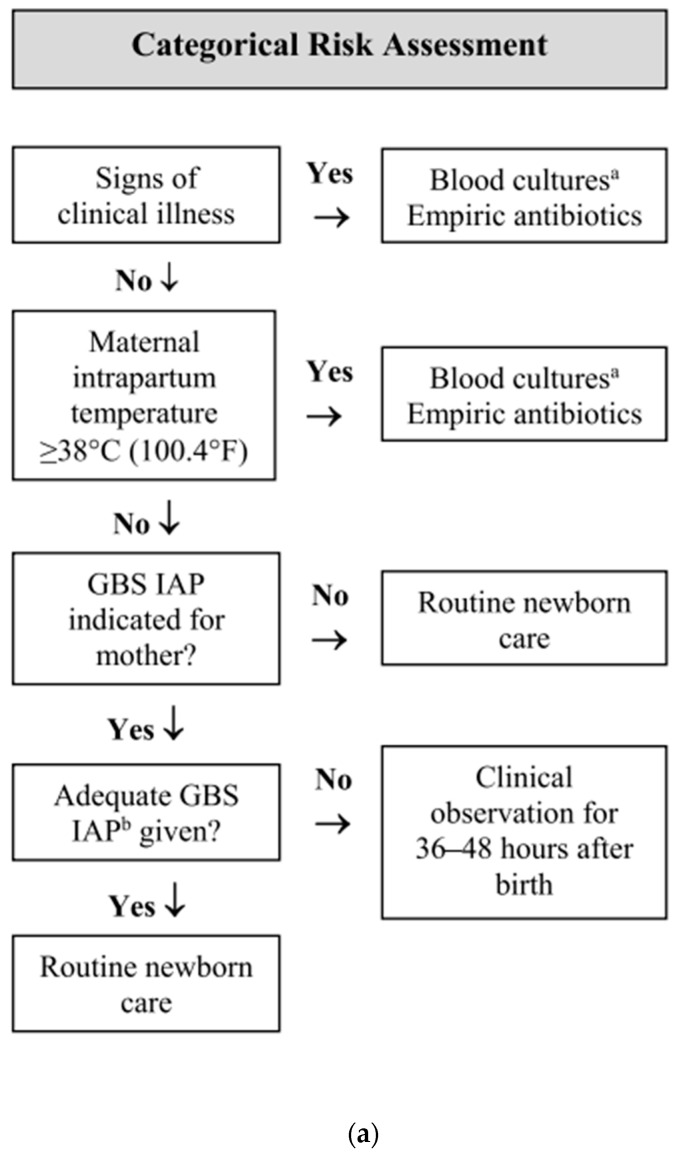

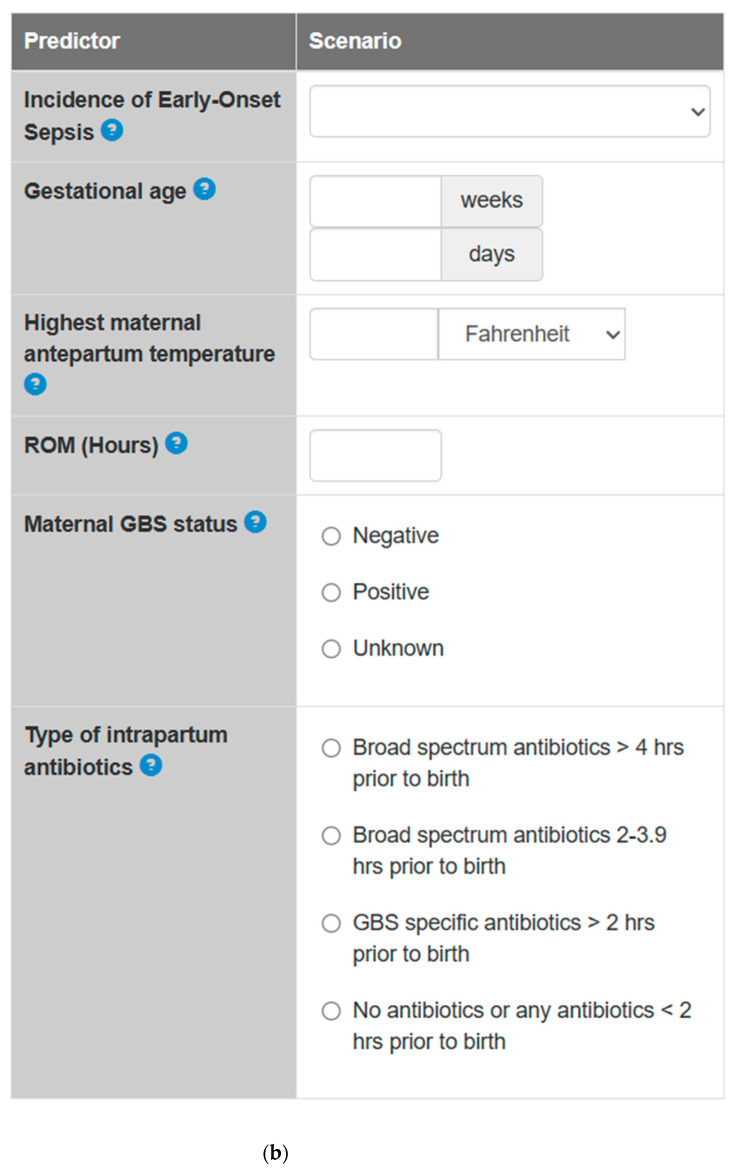

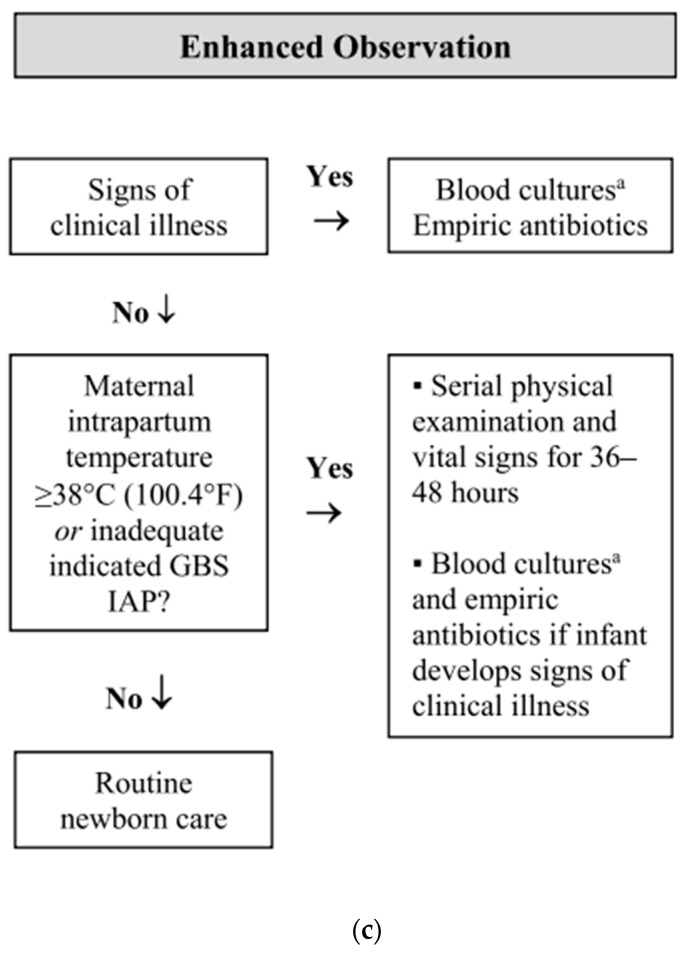
Options for EOS risk assessment among infants born at ≥35 weeks’ gestation. (**a**) Categorical risk assessment. (**b**) Neonatal Early-Onset Sepsis Calculator. (**c**) Enhanced observation (Adapted from [16]). ^a^ consider lumbar puncture ^b^ adequate GBS IAP is defined as the administration of penicillin G, ampicillin, or cefazolin ≥ 4 hours before delivery.

To date, diagnostic tests with the perfect ability to identify newborn infants likely to be infected are unavailable, and blood culture remains the gold diagnostic standard for EOS. Newborn infectious disease is manifested by clinical signs, but most are devious and difficult to distinguish from other conditions (e.g., transitional instability) [7,8,9,10,11,12,13]. Therefore, neonatal infectious disease remains a complex challenge for clinicians. In recent years, the so-called sepsis scores have been developed and validated, incorporating clinical and laboratory parameters [14]. Aiming to minimize antibiotic therapy and tailoring it to those who really need it, this new model estimates the probability of EOS using maternal risk factors associated with clinical signs in infants born at ≥34 weeks gestation [14]. This model is available as a Web-based Neonatal Early-Onset Sepsis Calculator (https://neonatalsepsiscalculator.kaiserpermanente.org, accessed on 23 April 2023) and includes recommended clinical actions at specific predicted risks. Figure 1 illustrates the three main approaches to identify neonates at risk for EOS. Although there is robust evidence that the sepsis calculator is able to reduce the use of antibiotics significantly [15], this strategy neither identifies with complete certainty all infants who will develop EOS nor completely avoids the treatment of infants who are not infected.

Our study aimed to compare the management of newborn infants at risk of EOS according to our unit guidelines and the Neonatal Early-Onset Sepsis Calculator’s recommendations retrospectively applied.

## 2. Results

A total of 1000 neonates born at ≥34 weeks’ gestational age (GA) during the study period were enrolled.

Demographic characteristics and risk factors are shown in Table 1.

According to our guidelines, 223/1000 (22.3%) newborns underwent blood investigations. On the contrary, following the EOS calculator’s recommendations, we would have performed blood tests just in 35/1000 newborns (3.5%) (*p* < 0.0001).

**Figure 2:**
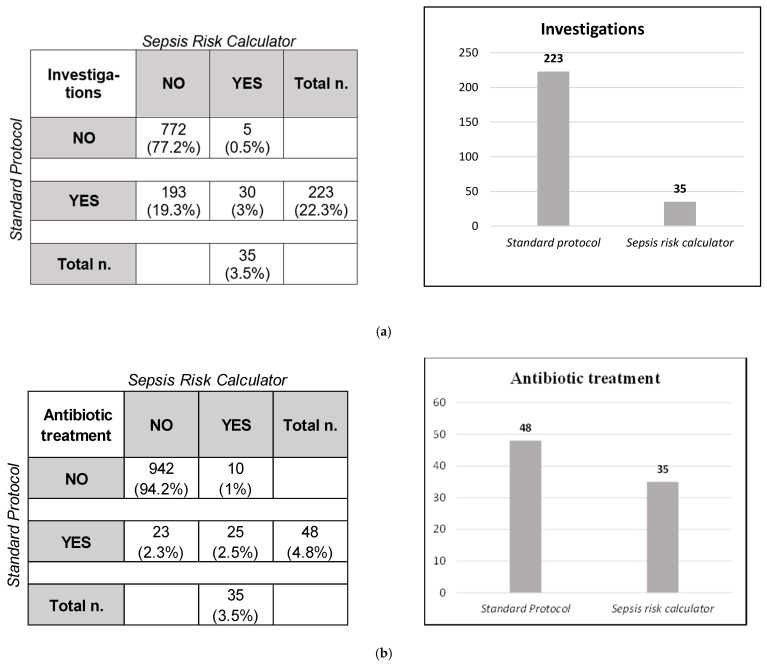
Comparison between our local guidelines and EOS calculator. (**a**) Newborns who underwent investigations. (**b**) Newborns who received antibiotic treatment.

According to Cohen’s kappa coefficient, the agreement between the two protocols was 0.18 (CI 95%: 0.12–0.24). Also, following the EOS calculator’s recommendations, we would not have performed the blood tests in 193 newborns (86.5% of this population). On the contrary, the blood tests would have been indicated in five newborns according to the EOS calculator but were not performed as per our protocol (Figure 2).

Using the standard protocol, 48/1000 infants received antibiotic therapy. Of these, two infants were equivocal, 25 presented with clinical illness (primarily respiratory distress), and 21 were asymptomatic. Moreover, 33.3% had a CRP between 15 and 30 mg/l, 29.2% had a CRP > 30 mg/L, and 18.8% had WBC > 25.000/mm^3^.

Based on the EOS calculator’s clinical features, 14 newborns were classified as “equivocal” and 35 as “clinical illness”. According to the EOS calculator, antibiotics were needed in those 35 infants with clinical illness.

The difference between the two protocols was not statistically significant (*p* = 0.12), indicating the same proportion of infants who underwent antibiotic therapy. The analysis showed that 25/48 infants were supposed to receive antibiotics according to both protocols, 23 according to our protocol and only ten according to the EOS calculator. These data were confirmed with a Cohen’s kappa coefficient of 0.58, proving moderate agreement (Figure 2).

Three newborns had a positive blood culture during the study period: one for *Enterococcus faecalis* and two for *Streptococcus agalactiae B (GBS).* All these infants never showed any clinical illness features reported in the EOS calculator and would not have needed antibiotic therapy according to the EOS calculator. The first case was a late preterm newborn who underwent blood investigations and culture without clinical signs and normal laboratory tests. The second case was a well-appearing newborn with bowel bleeding and a negative CRP; the third was an infant with max CRP of 33 mg/L without any clinical sign.

In a sub-population analysis of 18 newborns born to a mother with fever or Triple I suspected or confirmed, all newborns underwent blood investigations according to the standard protocol. In contrast, two would have undergone blood tests using the EOS calculator. Six of them (33%) received antibiotic therapy instead of two newborns with clinical illness that would have been treated according to the EOS calculator.

## 3. Materials and Methods

This was a single-centre observational retrospective study conducted at the Division of Neonatology at Filippo del Ponte Hospital in Varese, Italy.

**Figure 3:**
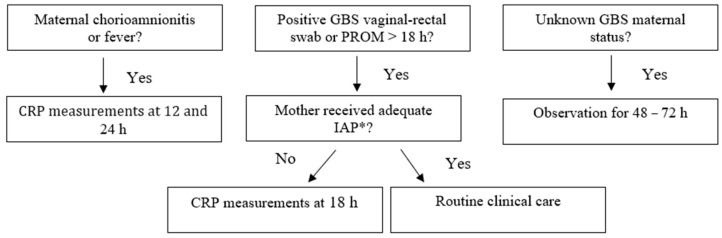
Management of neonates born at ≥37 weeks’ gestation according to our local guidelines (“Standard protocol”). * adequate IAP is defined as the administration of penicillin G, ampicillin, or cefazolin ≥ 4 h before delivery.

**Figure 4:**
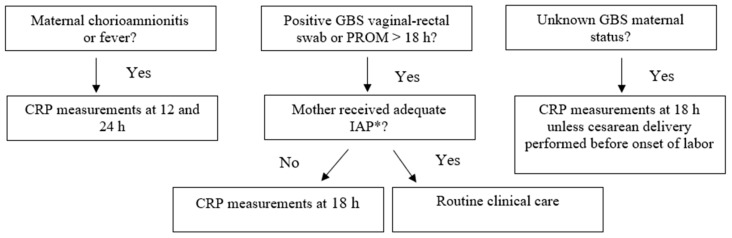
Management of neonates born at ≥34 and ≤36 + 6 weeks gestation according to our local guidelines (“Standard protocol”). * adequate IAP is defined as the administration of penicillin G, ampicillin, or cefazolin ≥ 4 h before delivery.

The first 1000 consecutive neonates born at ≥34 weeks’ gestation between January 2021 and May 2021 were enrolled. Outborn infants and those with major congenital anomalies were excluded from the study. According to our unit guidelines, neonates born at ≥34 weeks’ GA were managed differently based on risk factors and GA, as shown in Figure 3 and Figure 4. Antibiotic therapy is started when clinical signs of sepsis are present and/or C-reactive protein (CRP) is over 15 mg/L.

The duration of the antibiotic therapy is five days in those cases with CRP exceeding the cut-off level and a well-appearing baby with negative blood culture, seven days if the baby has symptoms and ten days in case of positive blood culture. Discontinuing antibiotic therapy for neonates with negative blood culture at 48 h, negative CRP and no clinical signs of sepsis are promoted. The first-line antibiotic therapy is intravenous ampicillin plus amikacin.

All healthy infants, even those who underwent therapy, were managed at the Nursery (Level I). Only infants with clinical signs and symptoms were transferred to the NICU (Level III) for continued care.

Retrospectively, the same babies were attributed EOS score to compare with our unit guidelines. Each newborn was classified as “well-appearing”, “equivocal” or “clinical illness” based on the characteristics reported in the EOS calculator website (https://neonatalsepsiscalculator.kaiserpermanente.org, accessed on 23 April 2023) [17]. Each patient’s EOS risk and subsequent management recommendation were determined using an EOS incidence rate of 0.5/1000 live births, in line with the data reported by the CDC [8,17].

Data collected included the following: neonatal data, mode of delivery and maternal and neonatal risk factors (GBS maternal status, amniotic membrane rupture ≥ 18 h, maternal fever > 38 °C and suspected or confirmed Triple I). We also described the presence and type of clinical signs of EOS, relevant laboratory results, blood culture and duration of antibiotic therapy.

Possible management recommendations were as follows: (1) no culture, no antibiotics, routine vitals; (2) no culture, no antibiotics, vitals every 4 h for 24 h; (3) blood culture, vitals every 4 h for 24 h; (4) strongly consider starting empiric antibiotics, vitals per NICU; and (5) empiric antibiotics, vitals per NICU.

Data were collected in a designated database, and a comparison between the different protocols was performed using McNemar’s test (statistical significance *p* < 0.05), and the Cohen’s kappa coefficient was used to assess the degree of agreement among.

## 4. Discussion

Avoiding unnecessary antibiotics remains one of the most critical challenges for neonatologists. However, early diagnosis and correct management of EOS remain crucial.

Early antibiotic exposure is associated with increased antibiotic resistance and intestinal microbiome impoverishment [18,19], and it has also been linked with late-onset sepsis, necrotizing enterocolitis, increased mortality and long-term health outcomes. Administration of antibiotics is also related to admission to the intensive care unit, invasive procedures and higher hospital costs. Of note, these aspects decrease the establishment of breastfeeding and rooming-in [20,21].

Two main approaches currently exist using risk factors to identify infants at increased risk of EOS: a categorical risk factor assessment and a multivariate risk assessment (Figure 1). The first is an algorithm for managing GBS-specific disease developed by the CDC that has been used as a general framework for preventing all EOS [8,22,23]. Limitations of this approach include a lack of clear definitions of newborn clinical illnesses, difficulties in identifying maternal chorioamnionitis, an inconsistent consideration of intrapartum antibiotics and a lack of neonatal cut-offs to define abnormal laboratory tests.

The second approach is a multivariate risk assessment based on the Kaiser Permanente EOS calculator developed to avoid overtreatment. The EOS calculator is based on a multivariate predictive risk model, intended to estimate a newborn’s individual risk of EOS by adding the perinatal and maternal risk factors and the infant’s clinical presentation [11,15,24]. This model overcomes some disadvantages of the CDC algorithm, such as the dichotomization of the continuous variables and the inclusion of maternal chorioamnionitis as an impactful risk factor for starting antibiotic therapy [25]. Several studies reported its efficacy in reducing antibiotic overtreatment, laboratory testing, painful procedures and NICU admissions [11,20,25].

Potential concern is the need for continuous monitoring to assess infants as clinically ill, equivocal or well appearing over the first 12–24 h after birth. Authors have also reported patients with culture-proven EOS who would not have received antibiotics based on the decision making of the EOS calculator [26,27,28].

Our local guidelines are based on the CDC algorithm with some amendments. First, we perform laboratory tests for all newborns born to mothers with inadequate IAP; second, we treat only newborns born to febrile mothers with clinical signs of sepsis or altered biomarkers such as CRP > 15 mg/L.

In our study, the laboratory tests could have been avoided in 86.5% of newborns according to the EOS calculator when compared to our local guidelines, resulting in a statistically significant difference in reducing unnecessary blood investigations with a relevant benefit in clinical practice. Potential advantages could have been less workload, more care for the newborns and less risk of incorrect or ambiguous evaluation. Our data are similar to those reported in a recent meta-analysis by Deshmukh et al. describing a reduction from 15.5% to 2.5% of laboratory tests using the EOS calculator [29]. In 2018, Dhudasia and Puopolo et al. confirmed a laboratory test reduction of 82% after introducing the EOS calculator [30]. Contrary to our unit’s protocol, only five newborns (0.6%) should have undergone blood investigations according to the EOS calculator. These babies needed transient respiratory support (high flow nasal-cannula or oxygen therapy) for mild respiratory distress that resolved in a few hours.

Another controversial aspect is the choice of biomarkers. According to our unit’s guidelines, the deciding factor to start antibiotics is CRP > 15 mg/L, as we know that CRP and PCT have a good negative predictive value but are neither sensitive nor specific to guide EOS care decisions in the absence of culture-confirmed infections. However, to date, serial evaluation of inflammatory markers is not recommended in well-appearing term newborns at risk of EOS [1,31].

In our study, the number of patients needing antibiotic therapy was substantially the same between the two approaches. However, the patients were not the same. According to the EOS calculator, just half of the newborns were eligible for antibiotic treatment, and ten more infants should have started therapy based on respiratory distress. Conversely, in our unit, infants with isolated respiratory distress with negative biomarkers undergo a close clinical observation and are not administered antibiotics immediately.

According to our experience, the EOS calculator seems at risk of overestimating respiratory symptoms that can overlap with other neonatal clinical manifestations, such as the transient tachypnea of newborns. In accordance with Laccetta and colleagues, careful consideration of the risk factors for EOS, anamnestic data and alternative diagnoses should further reduce unnecessary antibiotics administration. For equivocal patients (presenting with tachycardia, tachypnea, temperature instability or respiratory distress), other clinical indicators of EOS, such as skin discolouration and circulatory signs, altered behaviour or responsiveness, and feeding difficulties should be considered [32].

In our study, we found that the EOS calculator failed to detect three confirmed neonatal bloodstream infections. However, the correct interpretation of these cases is questionable, and all of them had no clinical signs suggesting a transient bacteremia or a benign course due to early therapy.

Currently, in high-income countries, the incidence of EOS in term and late preterm babies has reduced to around 0.5–1/1000 and does not differ from our hospital data in the last years. Thus, according to the EOS calculator, in a small population of 18 infants born to mothers with fever, blood tests and antibiotics would have been needed in two infants. However, according to our guidelines, they all underwent blood tests and six newborns were started on antibiotics. Although the local EOS incidence of 0.5/1000 live births was set up in the calculator, it is reasonable that a higher incidence was assumed in this sub-population, and the new definition of “Triple I” was incorporated [33].

An interesting approach is the risk assessment of newborn clinical conditions suggested by the GBS Prevention Working Group of Emilia-Romagna (Italy) using serial physical examination (SPE) performed at standardized time points for all newborns. This approach, however, requires experience and specific staff training [34,35].

A promising new approach has been recently published. It includes the combination of EOS calculator and SPE for all newborns with the possibility of evaluating the administration of antibiotic therapy in infants showing only mild respiratory symptoms with other systemic signs. Combined strategy seems to significantly reduce laboratory testing and antibiotic prescriptions [36].

Our study has some limitations. First, it was a retrospective study with a small sample size and the EOS calculator was applied retrospectively. Also, the overall incidence of presumed culture-proven EOS in the studied population (3/1000) is likely to be overestimated because of the limited study period.

## 5. Conclusions

Although the use of EOS has dramatically decreased the number of babies undergoing multiple blood examinations after birth, there is still room for further improvements in evaluating babies at risk of EOS to avoid missing asymptomatic bloodstream infections and to reduce antibiotic administration.

The missing cases of three bloodstream infections deserve careful consideration. This might lead to the implementation of clinical indicators to be considered in the calculator, such as altered behaviour or responsiveness, feeding difficulties, altered skin colour, body temperature, acidosis and glycemic instability.

Further randomized studies evaluating the safety and implementation of EOS calculator are urgently warranted.

## Figures and Tables

**Table 1:** Demographic characteristics and risk factors for EOS among the study population.

	n°	%
**Sex**		
Male	515	51.5%
Female	485	48.5%
**Growth**		
AGA	858	85.8%
SGA	109	10.9%
LGA	33	3.3%
**Mode of delivery**		
Vaginal delivery	796	79.6%
Cesarean section	204	20.4%
**Maternal GBS status**		
Negative	721	72.1%
Unknown	120	12%
Positive	159	15.9%
**Positive GBS status and adequate IAP**	55	34.6% ^1^
**Positive GBS status and inadequate IAP**	92	57.9% ^1^
**Duration of ROM**		
ROM < 18 h	904	90.4%
ROM > 18 h	96	9.6%
**PROM > 18 h and antibiotics**	57	59.4% ^2^
**PROM > 18 h and no antibiotics**	39	40.6% ^2^
**Triple I**		
Confirmed Triple I	9	0.9%
Suspected Triple I	2	0.2%
Isolated fever	4	0.4%

^1^ Considered on total population with maternal GBS status positive. ^2^ Considered on total population with ROM > 18.

## Data Availability

The data presented in this study are available on request from the corresponding author.
